# Enhanced recovery after thoracic surgery: Clinical outcomes and imaging-based evaluation of a multimodal rehabilitation strategy

**DOI:** 10.1097/MD.0000000000047294

**Published:** 2026-01-30

**Authors:** Xinye Li, Zhenjun Hu, Aotian Guo, Zhengfu He

**Affiliations:** a Department of Thoracic Surgery, Sir Run Run Shaw Hospital, School of Medicine, Zhejiang University, Hangzhou City, Zhejiang Province, China; b Department of TCM, Yingfeng Community Health Service Center, Zhejiang Xiaoshan Hospital, Hangzhou, Hangzhou City, Zhejiang Province, China.

**Keywords:** enhanced recovery after surgery (ERAS), health-related quality of life, imaging assessment, postoperative complications, pulmonary function, video-assisted thoracoscopic surgery (VATS)

## Abstract

Enhanced recovery after surgery (ERAS) protocols are increasingly adopted in thoracic surgery to optimize recovery, but evidence integrating surgical outcomes with imaging-based postoperative recovery assessment remains limited, particularly for video-assisted thoracoscopic surgery. This single-center retrospective cohort study included patients undergoing elective video-assisted thoracoscopic surgery, divided into an ERAS group and a conventional care group. Outcomes analyzed included clinical indices (postoperative complications, chest tube duration, hospital stay), numerical rating scale pain scores, pulmonary function (forced expiratory volume in 1 second, forced vital capacity), health-related quality of life (European Organization for Research and Treatment of Cancer Quality of Life Questionnaire-Core 30), and imaging assessments (computed tomography for lung re-expansion rate, chest radiography for inflammatory resolution rate). Compared with the conventional group, the ERAS group had a significantly lower total postoperative complication rate (31.2% vs 71.9%, *P* < .01), with significantly fewer cases of atelectasis and pleural effusion. The ERAS group also had significantly shorter chest tube duration and postoperative hospital stay, better pain control (numeric rating scale scores at 1 or 3 days postoperatively, both *P* < .001), significantly superior pulmonary function at 1 week and 1 month, significantly higher 1 week lung re-expansion rate (89.2 ± 5.3% vs 72.6 ± 4.5%, *P* < .001) and 1 month inflammatory resolution rate (84.7 ± 5.9% vs 68.0 ± 5.6%, *P* < .001), and significantly improved 1 month European Organization for Research and Treatment of Cancer Quality of Life Questionnaire-Core 30 scores (*P* < .001). A multimodal ERAS strategy significantly enhances postoperative outcomes in patients undergoing video-assisted thoracoscopic surgery. Imaging-based evaluation provides objective evidence of lung recovery, supporting its role as a valuable adjunct to clinical and functional assessments in thoracic surgical practice.

## 1. Introduction

Enhanced recovery after surgery (ERAS) has evolved as a multidisciplinary perioperative care framework designed to mitigate surgical stress responses and accelerate functional restoration, with well-established efficacy across multiple surgical specialties.^[1,2]^ In thoracic surgery, the adoption of ERAS protocols has grown steadily over the past decade, driven by accumulating evidence of improved patient outcomes.^[3]^ A study by Van Haren et al demonstrated that ERAS implementation reduced pulmonary and cardiac complications following thoracotomy for lung cancer, highlighting its potential to address the inherent risks of thoracic interventions.^[4]^ This benefit was further validated by a 2021 retrospective analysis of single-port video-assisted thoracoscopic surgery (VATS), which found that ERAS-aligned practices such as non-indwelling chest drains significantly alleviated postoperative pain and shortened hospital stays.^[5]^ A retrospective observational study by Marques et al has reinforced this finding: consistent reductions in morbidity were identified across surgical populations,^[6]^ while a 2024 update focusing specifically on thoracic surgery confirmed that ERAS protocols consistently shorten length of stay and likely reduce complication rates.^[7]^

VATS has emerged as the first-line minimally invasive approach for lung resections and mediastinal procedures,^[8]^ offering advantages of reduced surgical trauma and faster initial recovery compared to open thoracotomy.^[9]^ Despite these merits, VATS still remains barriers to optimal postoperative outcomes by its specific challenges, including persistent chest wall pain, delayed lung re-expansion, and subclinical atelectasis.^[10]^ Bertolaccini et al analysis of a national VATS database noted that even with technical refinements, postoperative pain management and respiratory rehabilitation remain unmet needs in routine clinical practice.^[11]^ This gap underscores the need for tailored ERAS strategies that integrate intraoperative and postoperative interventions to amplify the minimally invasive benefits of VATS. A 2022 retrospective cohort study evaluating ERAS in robotic and VATS lobectomy found that ERAS implementation independently reduced hospital stays, with synergistic benefits when combined with minimally invasive techniques.^[12]^ Similarly, targeted nerve blocks as a key ERAS component in VATS patients, was found to demonstrate with reduced intraoperative anesthetic requirements and improved postoperative pain control, emphasizing the value of ERAS optimization.^[13]^

Despite the growing body of evidence supporting thoracic ERAS, conventional outcome evaluation remains limited by overreliance on subjective clinical indices and functional assessments.^[14]^ Patient-reported pain scores, for example, often fail to correlate with objective tissue healing,^[15]^ while spirometric measures of pulmonary function cannot capture subclinical anatomical abnormalities such as small-volume pleural effusions or focal atelectasis.^[16]^ According to Kang et al, the limitations of subjective recovery metrics were highlighted among a group of esophageal resection patients, noting that clinical assessments alone missed subclinical anastomotic issues later identified by imaging.^[17]^ This disconnect is particularly relevant in VATS patients, where subtle impairments in lung re-expansion may precede clinical symptoms. According to Chung et al, the standardized imaging follow-up after aortic surgery further illustrated the clinical value of objective anatomical assessment, with imaging-detected abnormalities prompting timely reintervention in 9% of patients who would have otherwise been missed by clinical monitoring alone.^[18]^ Despite these insights, the systematic integration of imaging into ERAS outcome evaluation for thoracic surgery remains understudied, as noted in a 2024 consensus statement on ERAS implementation priorities, which identified “objective recovery markers” as a critical unmet need.^[14]^

Chest computed tomography (CT) and radiography offer unique capabilities for quantitative assessment of thoracic surgical recovery, including lung re-expansion, inflammatory resolution, and pleural space status. A 2021 study of COVID-19-related acute respiratory distress syndrome demonstrated that CT can accurately quantify lung re-expansion with high reproducibility, a technique adaptable to postoperative VATS patients.^[19]^ Otake et al further showed in 2024 that CT-derived measurements of lung expansion correlate with long-term pulmonary function, suggesting potential as a prognostic tool.^[20]^ However, few studies have systematically linked these imaging metrics to ERAS interventions. A 2022 review of thoracic ERAS outcomes found that just a few studies included objective imaging endpoints, with most focusing solely on clinical or functional outcomes.^[21]^ This gap is problematic given that anatomical recovery has been identified as a key mediator of long-term quality of life in lung resections.^[22]^

The present study addresses these limitations by evaluating the efficacy of a multimodal ERAS strategy specifically tailored to VATS patients, with a focus on integrating intraoperative optimization and imaging-based outcome assessment. Building on prior work by Sedighim et al, who emphasized the need for personalized perioperative care in minimally invasive thoracic surgery,^[23]^ we designed our ERAS protocol to include targeted analgesia, structured respiratory training, and early mobilization, which was built and supported by the ERAS/ESTS clinical practice guidelines.^[24]^ By incorporating CT quantitative analysis of lung re-expansion and inflammatory resolution, we aim to provide objective evidence of ERAS-mediated recovery, complementing traditional clinical and functional assessments. This study also attempts to validate a synergistic approach to VATS perioperative care that combines minimally invasive surgery with tailored ERAS interventions and robust imaging assessment, advancing the evidence base for thoracic surgical recovery optimization.

## 2. Materials and methods

### 2.1. Study design and ethics

#### 2.1.1. Design type

This study adopted a single-center, retrospective cohort design. The design was selected to evaluate the real-world efficacy of a multimodal ERAS strategy in patients undergoing VATS, as retrospective cohort studies are well-suited for analyzing outcomes of established clinical practices while minimizing selection bias from prospective randomization. This approach aligned with the Strengthening the Reporting of Observational Studies in Epidemiology (STROBE) statement recommendations for observational research, which emphasize transparency in data collection and outcome assessment to ensure scientific rigor.

#### 2.1.2. Study period and setting

The study was conducted at the Department of Thoracic Surgery, Sir Run Run Shaw Hospital, School of Medicine, Zhejiang University – a tertiary referral center with specialized expertise in minimally invasive thoracic procedures – between January 2024 and June 2024. This 6-month period was chosen to accumulate a sufficient sample size while ensuring consistency in surgical techniques and perioperative care protocols, as the hospital’s VATS and ERAS practices were standardized during this timeframe.

#### 2.1.3. Ethics approval

The study was approved by the Institutional Review Board of Sir Run Run Shaw Hospital, School of Medicine, Zhejiang University. Written informed consent for participation and data use was obtained from all patients or their family members prior to surgery. All patient information was de-identified to protect privacy, such as removing names and medical record numbers. The study was conducted in accordance with the ethical standards of the 2013 Declaration of Helsinki.

### 2.2. Study population

#### 2.2.1. Inclusion criteria

Eligible patients met the following criteria: age between 18 and 80 years, to exclude pediatric patients and elderly individuals with extreme frailty that may confound recovery outcomes; undergoing elective VATS, including lung lobectomy, lung wedge resection, or mediastinal tumor resection – these procedures were selected as they represent the most common minimally invasive thoracic surgeries and align with the focus on VATS in the study’s research objective; preoperative forced expiratory volume in 1 second (FEV1) ≥ 60% of the predicted value, to ensure patients had sufficient baseline lung function to tolerate surgery and complete postoperative follow-up; and complete clinical and imaging follow-up at 1 week and 1 month postoperatively, to enable comprehensive assessment of short- and medium-term recovery, as outlined in the study’s outcome framework.

#### 2.2.2. Exclusion criteria

Patients were excluded if they: required emergency surgery, as emergency procedures are associated with higher perioperative stress and confounding factors such as acute illness that would interfere with evaluating ERAS efficacy; had severe comorbidities, including end-stage chronic obstructive pulmonary disease or New York Heart Association Class IV heart failure, as these conditions independently increase postoperative complication risk and may mask the effect of ERAS interventions; had malignant tumors with distant metastasis, as metastatic disease introduces additional treatment-related variables that could impact recovery; and were readmitted within 1 week postoperatively, as unplanned readmission indicates acute complications that would disrupt standardized follow-up and outcome measurement.

### 2.3. Group allocation and interventions

Patients were divided into 2 groups based on perioperative care protocols implemented during their hospitalization (non-randomized, per clinical practice patterns).

#### 2.3.1. ERAS group (multimodal strategy)

The ERAS group received a structured, multimodal intervention tailored to VATS patients, informed by the 2022 Clinical Practice Guidelines for Enhanced Recovery After Thoracic Surgery from the European Society of Thoracic Surgeons (ESTS).^[24]^ Specific components included.

##### 2.3.1.1. Perioperative analgesia

Intraoperative intercostal nerve block (targeting T3–T8 intercostal spaces) with 0.5% ropivacaine (2–3 mL/space), followed by postoperative intravenous patient-controlled analgesia (PCA) with fentanyl (loading dose 1–2 μg/kg, bolus dose 10 μg, lockout time 10 minutes). This regimen was selected to provide targeted pain control, reducing opioid consumption and opioid-related side effects such as respiratory depression that may hinder recovery.

##### 2.3.1.2. Respiratory training

Initiated 3 days preoperatively and continued until 1 week postoperatively, with 3 sessions/d (15 minutes/session) led by certified respiratory therapists. Training included diaphragmatic breathing (with visual feedback via a spirometer) and effective coughing (to clear secretions without excessive chest wall strain), aiming to preserve respiratory muscle function and promote lung re-expansion.

##### 2.3.1.3. Early mobilization

Standardized progression of activity: bed exercises such as ankle pumps and shoulder rolls, initiated 6 hours postoperatively; ambulation of ≥50 m within 24 hours postoperatively; and ambulation of ≥200 m/d by 48 hours postoperatively. Early mobilization was designed to reduce complications such as deep vein thrombosis and atelectasis, and accelerate functional recovery.

##### 2.3.1.4. Nutrition support

Individualized care guided by the Nutrition Risk Screening 2002 (NRS-2002) score.^[25]^ Patients with an NRS-2002 score ≥ 3 (indicating moderate-to-high nutrition risk) received oral nutritional supplements (200–400 kcal/d) starting from the first postoperative day, to maintain energy stores and support tissue repair.

#### 2.3.2. Conventional group

The conventional group received standard perioperative care without structured ERAS components, reflecting pre-ERAS institutional practices.

##### 2.3.2.1. Perioperative analgesia

Postoperative intravenous PCA with fentanyl alone (loading dose 2–3 μg/kg, bolus dose 15 μg, lockout time 10 minutes), without intraoperative nerve block. This regimen relied solely on systemic opioids, consistent with traditional pain management for thoracic surgery.

##### 2.3.2.2. Respiratory training

Ad-hoc guidance such as verbal instructions on deep breathing was provided by nursing staff postoperatively, with no standardized pre- or postoperative training schedule or oversight by respiratory therapists.

##### 2.3.2.3. Early mobilization

Delayed activity progression: bed exercises initiated 24 hours postoperatively; ambulation of ≥50 m allowed only after 48 hours postoperatively. This delay aligned with historical concerns about early activity increasing postoperative pain or complications.

##### 2.3.2.4. Nutrition support

Routine oral nutrition, with oral intake resumed based on patient tolerance (typically 24–48 hours postoperatively). No formal nutritional risk assessment was performed, and nutritional supplements were not systematically prescribed.

### 2.4. Surgical protocol

#### 2.4.1. Standardized VATS technique

All VATS procedures were performed by the same team of 3 attending thoracic surgeons with ≥5 years of specialized experience in minimally invasive thoracic surgery, ensuring consistency in surgical technique. The standardized protocol included.

##### 2.4.1.1. Anesthesia management

Lung-protective ventilation (tidal volume 6–8 mL/kg ideal body weight, positive end-expiratory pressure 5–8 cm H₂O, fraction of inspired oxygen [FiO₂] ≤ 0.6) to minimize perioperative lung injury.

##### 2.4.1.2. Surgical technique

For lung resections, hilar dissection and vessel ligation were performed using endoscopic staplers (Ethicon Endo-Surgery, Blue Ash); for mediastinal tumors, blunt and sharp dissection was used to isolate and resect lesions. Hemostasis was achieved using an ultrasonic scalpel (Ethicon Harmonic ACE+, Johnson & Johnson, Cincinnati) to reduce intraoperative bleeding and tissue trauma.

##### 2.4.1.3. Chest tube management

A 28-Fr chest tube was placed in the pleural space for drainage. Chest tubes were removed when daily drainage volume was <200 mL, no air leak was detected for ≥24 hours, and chest imaging (chest radiograph or CT) confirmed no significant pleural effusion or atelectasis – this removal criterion aligned with the 2019 guidelines for chest tube management in thoracic surgery from The Annals of Thoracic Surgery.

#### 2.4.2. Recorded surgical indices

Key surgical variables were extracted from operative records and anesthesia charts: operation time (defined as the duration from skin incision to skin closure); intraoperative blood loss (measured via suction canister volume and gauze weighing, with 1 g gauze weight equivalent to 1 mL blood); chest tube duration (calculated as the number of days from chest tube placement to removal). These indices were recorded to confirm that surgical factors were balanced between groups, ensuring that differences in outcomes could be attributed to ERAS interventions rather than surgical variability.

### 2.5. Outcome measures

#### 2.5.1. Clinical outcomes

Clinical outcomes focused on core markers of postoperative recovery – postoperative hospital stay: calculated as the number of days from the date of surgery to the date of discharge, with discharge defined as the time when patients met clinical stability criteria such as no fever, adequate pain control and ability to ambulate independently. Complication rates: including atelectasis, pleural effusion requiring drainage, and pneumonia. Complications were diagnosed based on a combination of clinical symptoms, laboratory tests, and confirmatory imaging, using the 2022 Society of Thoracic Surgeons definitions for thoracic surgical complications to ensure consistency.

#### 2.5.2. Pain assessment

Postoperative pain was evaluated using the numeric rating scale (NRS), a validated tool for quantifying pain intensity (0 = no pain, 10 = worst imaginable pain). NRS scores were recorded at 1 and 3 days postoperatively, during morning rounds by trained nursing staff who were blinded to the study’s research hypotheses. Blinding was implemented to reduce bias in pain assessment, as staff awareness of group allocation could influence score recording.

#### 2.5.3. Pulmonary function

Pulmonary function was assessed using a portable spirometer (MasterScreen Pneumo, Jaeger, Germany) at 1 week and 1 month postoperatively. Measured parameters included FEV1 (absolute volume in liters) and forced vital capacity (FVC, absolute volume in liters), with values also reported as percentages of the predicted value. Predicted values were calculated using the 2019 European Respiratory Society (ERS) reference equations, which account for age, gender, height, and weight to adjust for individual differences in lung function. All spirometric tests were performed by certified respiratory technicians following the standardized procedure outlined in the 2019 American Thoracic Society/ERS (ATS/ERS) guidelines for lung function testing, ensuring reproducibility across time points.

#### 2.5.4. Imaging outcomes

**Figure 1.**
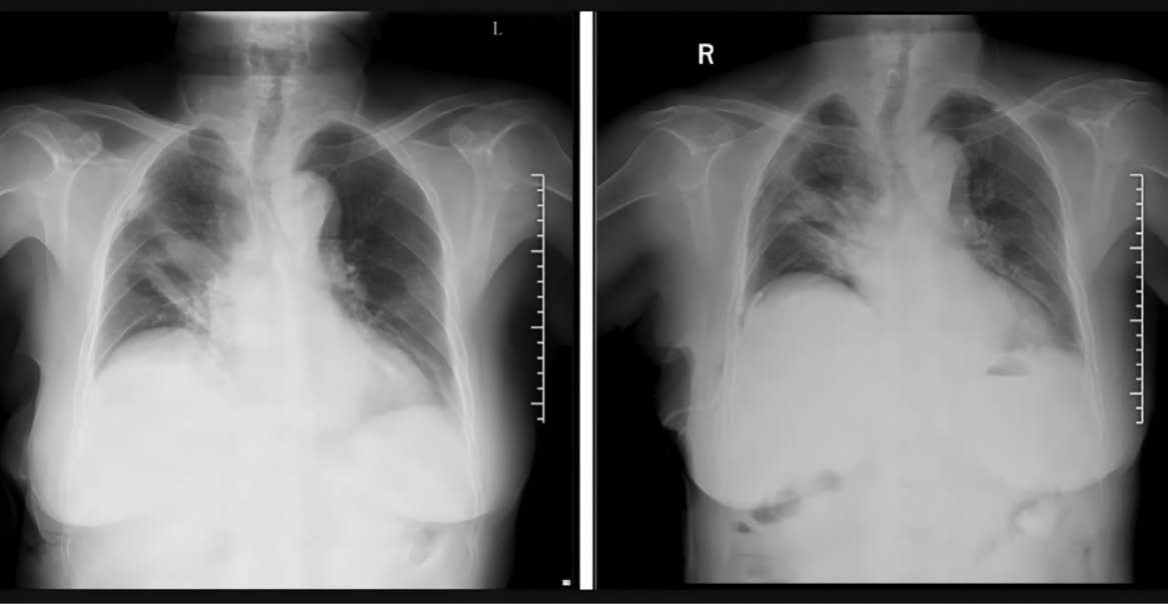
Comparison of postoperative 1 week lung re-expansion via chest CT between the ERAS group and conventional group. Left panel: Chest CT of a patient in the ERAS group, showing uniform lung field density and only a tiny focal non-reexpanded region. Right panel: Chest CT of a patient in the conventional group, showing obvious focal hyperdense shadows in the lung fields and multiple non-reexpanded regions. CT = computed tomography, ERAS = enhanced recovery after surgery.

Imaging-based evaluation was a core component of outcome assessment (Fig. 1). All imaging was reviewed by 2 independent radiologists with 5 and 8 years of thoracic imaging experience, who remained blinded to patient group allocation; for the 1 week lung re-expansion rate, assessment was performed via chest CT (64-slice scanner, Siemens Somatom Definition Flash) using a semiautomated software tool (Syngo.via, Siemens Healthineers), with the re-expansion rate calculated as the percentage of re-aerated lung tissue relative to the total lung volume, while 1 week pleural effusion volume was categorized based on CT findings into 3 grades (1: < 50 mL, minimal; 2: 50–200 mL, moderate; 3: > 200 mL, large) – a grading system aligned with standard thoracic imaging terminology to ensure consistency – and for the 1 month inflammatory resolution rate, assessment was conducted via chest radiography, where resolution was defined as >70% reduction in the extent of postoperative pulmonary infiltrates (compared to postoperative day 1 radiographs), disagreements between radiologists were resolved via consensus, and inter-observer agreement was quantified using the kappa statistic.

#### 2.5.5. Quality of life

Health-related quality of life (HRQOL) was assessed at 1 month postoperatively using the European Organization for Research and Treatment of Cancer Quality of Life Questionnaire-Core 30 (EORTC QLQ-C30, version 3.0) – a validated, widely used instrument for measuring quality of life in surgical and oncology populations. The questionnaire includes 2 key domains: global quality of life score (range 0–100, higher scores indicating better overall quality of life) and physical function subscale (range 0–100, higher scores indicating better ability to perform daily activities). The EORTC QLQ-C30 has demonstrated good reliability (Cronbach’s α > 0.70) and validity in thoracic surgical patients.

### 2.6. Statistical analysis

All statistical analyses were performed using SPSS software (version 26.0, IBM Corp., Armonk) for data management and hypothesis testing. Data were presented based on variable type: Continuous variables (e.g., age, body mass index (BMI), operation time, intraoperative blood loss, pulmonary function indices FEV1 and FVC, NRS pain scores, lung re-expansion rate, inflammatory resolution rate, and EORTC QLQ-C30 scores) were reported as mean ± standard deviation. Categorical variables (e.g., gender, preoperative comorbidities, surgical procedure type, and postoperative complication types) were reported as numbers with percentages to describe group characteristics and outcome frequencies.

Group comparisons were performed using appropriate statistical tests based on variable type: Independent samples *t*-test was used to compare the continuous variables between the ERAS and conventional groups. χ^2^ test was used for categorical variables.

## 3. Results

### 3.1. Baseline characteristics

As presented in Table 1, no significant differences were observed between the ERAS group and the conventional group across all baseline variables, supporting the validity of subsequent outcome comparisons. Demographic characteristics were well balanced, including age, gender, and BMI. The mean age was 53.2 ± 6.2 years in the ERAS group and 55.2 ± 6.1 years in the conventional group. Male patients accounted for 53.1% of the ERAS group and 43.8% of the conventional group. The mean BMI was 23.6 ± 1.6 kg/m² and 24.0 ± 1.6 kg/m², respectively.

**Table 1:** Baseline characteristics of patients undergoing video-assisted thoracoscopic surgery (VATS): comparisons between the ERAS group and conventional group.

Variable	ERAS group (n = 32)	Conventional group (n = 32)	Statistical method and *P* value
Demographic characteristics			
Age, yr	53.2 ± 6.2	55.2 ± 6.1	Independent samples *t*-test, *P* > .05
Gender, n (%)			
Male	17 (53.1%)	14 (43.8%)	χ^2^ test, *P* > .05
Female	15 (46.9%)	18 (56.3%)	χ^2^ test, *P* > .05
BMI, kg/m²	23.6 ± 1.6	24.0 ± 1.6	Independent samples *t*-test, *P* > .05
Preoperative clinical variables			
Comorbidities, n (%)			
Hypertension (History = 1)	3 (9.4%)	0 (0.0%)	χ^2^ test, *P* > .05
Diabetes (History = 2)	2 (6.2%)	4 (12.5%)	χ^2^ test, *P* > .05
Chronic bronchitis (History = 3)	2 (6.2%)	2 (6.2%)	χ^2^ test, *P* > .05
Coronary heart disease (History = 4)	3 (9.4%)	1 (3.1%)	χ^2^ test, *P* > .05
Multiple comorbidities	6 (18.8%)	11 (34.4%)	χ^2^ test, *P* > .05
No comorbidities (History = 0)	16 (50.0%)	14 (43.8%)	χ^2^ test, *P* > .05
Preoperative pulmonary function			
FEV1, L	3.07 ± 0.557	2.97 ± 0.565	Independent samples *t*-test, *P* > .05
FEV1, % predicted	91.1 ± 3.0%	90.9 ± 3.2%	Independent samples *t*-test, *P* > .05
FVC, L	3.55 ± 0.808	3.459 ± 0.8167	Independent samples *t*-test, *P* > .05
FVC, % predicted	91.3 ± 3.5%	91.1 ± 3.7%	Independent samples *t*-test, *P* > .05
Surgical procedure type, n (%)			
VATS lung lobectomy	10 (31.2%)	13 (40.6%)	χ^2^ test, *P* > .05
VATS lung wedge resection	14 (43.8%)	12 (37.5%)	χ^2^ test, *P* > .05
VATS mediastinal tumor resection	8 (25.0%)	7 (21.9%)	χ^2^ test, *P* > .05

BMI = body mass index, ERAS = enhanced recovery after surgery, FEV1= forced expiratory volume in 1 second, FVC = forced vital capacity, VATS = video-assisted thoracoscopic surgery.

Preoperative comorbidities and pulmonary function parameters also showed no significant intergroup differences. The prevalence of hypertension was 9.4% in the ERAS group and 0.0% in the conventional group, while diabetes occurred in 6.2% and 12.5%, respectively. Regarding pulmonary function, the mean FEV₁ was 3.07 ± 0.557 L in the ERAS group and 2.97 ± 0.565 L in the conventional group. The mean FVC was 3.55 ± 0.808 L and 3.46 ± 0.817 L, respectively.

Furthermore, the distribution of surgical procedures was comparable between the 2 groups. The proportion of VATS lobectomy was 31.2% in the ERAS group and 40.6% in the conventional group, while VATS wedge resection accounted for 43.8% and 37.5%, respectively. This balance minimizes potential confounding effects arising from baseline disparities and ensures that any postoperative differences observed can be attributed primarily to the ERAS intervention rather than preexisting variations.

### 3.2. Surgical and clinical outcomes

#### 3.2.1. Intraoperative and postoperative surgical variables

As shown in Table 2, intraoperative parameters were comparable between the ERAS and conventional groups, including operation time and intraoperative blood loss. The mean operation time was 107.22 ± 13.22 minutes in the ERAS group and 112.19 ± 13.27 minutes in the conventional group. The mean intraoperative blood loss was 78.13 ± 18.78 mL in the ERAS group and 83.28 ± 19.08 mL in the conventional group. These findings indicate that implementation of the ERAS strategy did not compromise surgical efficiency or technical consistency.

**Table 2:** Intraoperative and postoperative surgical-related variables in the ERAS group and conventional group.

Variable	ERAS group (n = 32)	Conventional group (n = 32)	Statistical method and *P* value
Intraoperative variables			
Operation time, min	107.22 ± 13.22	112.19 ± 13.27	Independent samples *t*-test, *P* > .05
Intraoperative blood loss, mL	78.13 ± 18.78	83.28 ± 19.08	Independent samples *t*-test, *P* > .05
Postoperative surgical-related variables			
Chest tube duration, d	2.84 ± 0.68	5.44 ± 1.05	Independent samples *t*-test, *P* < .01
Postoperative hospital stay, d	4.50 ± 0.76	7.38 ± 1.13	Independent samples *t*-test, *P* < .01

ERAS = enhanced recovery after surgery.

In contrast, the ERAS group exhibited clear advantages in postoperative recovery. The mean chest tube duration was significantly shorter in the ERAS group (2.84 ± 0.68 days) than in the conventional group (5.44 ± 1.05 days). Similarly, the mean postoperative hospital stay was reduced in the ERAS group (4.50 ± 0.76 days) compared with the conventional group (7.38 ± 1.13 days). Both differences were statistically significant (*P* < .01).

A shorter chest tube duration not only minimizes patient discomfort but also decreases the risk of tube-related complications such as infection and dislodgment, thereby facilitating early ambulation. Likewise, a reduced hospital stay alleviates the burden on healthcare resources such as bed occupancy and nursing workload, while allowing patients to resume normal activities sooner.

#### 3.2.2. Postoperative pain scores

Postoperative pain intensity, evaluated using the numerical rating scale (NRS), differed significantly between the 2 groups at both postoperative day 1 and day 3 (Table 3). On postoperative day 1, the mean NRS score was 2.34 ± 0.48 in the ERAS group and 5.06 ± 0.88 in the conventional group, with a statistically significant difference (independent samples *t*-test, *P* < .001). On postoperative day 3, the mean NRS score remained lower in the ERAS group (1.31 ± 0.47) than in the conventional group (3.06 ± 0.88), and this difference also reached statistical significance (*P* < .001).

**Table 3:** Postoperative pain scores assessed by numerical rating scale (NRS) in the ERAS group and conventional group.

Time point postoperatively	ERAS group (n = 32)	Conventional group (n = 32)
1 d	2.34 ± 0.48	5.06 ± 0.88
	Independent samples *t*-test, *P* < .001	
3 d	1.31 ± 0.47	3.06 ± 0.88
	Independent samples *t*-test, *P* < .001	

ERAS = enhanced recovery after surgery, NRS = numerical rating scale.

#### 3.2.3. Postoperative complication rates

The ERAS group demonstrated a substantial reduction in the overall postoperative complication rate compared with the conventional group (Table 4). The total complication rate was 31.2% in the ERAS group and 71.9% in the conventional group, representing a statistically significant difference (*P* < .01).

**Table 4:** Postoperative complication rates (atelectasis, pleural effusion, pneumonia) in the ERAS group and conventional group.

Complication category	ERAS group (n = 32), n (%)	Conventional group (n = 32), n (%)	Statistical method and *P* value
1. Individual complications			
Atelectasis (type 1)*	5 (15.6%)	12 (37.5%)	χ^2^ test, *P* < .05
Pleural effusion (type 2)†	7 (21.9%)	14 (43.8%)	χ^2^ test, *P* < .05
Pneumonia (type 3)‡	6 (18.8%)	9 (28.1%)	χ^2^ test, *P* > .05
2. Overall complications§			
Total complication cases	10 (31.2%)	23 (71.9%)	χ^2^ test, *P* < .01
3. No complication cases	22 (68.8%)	9 (28.1%)	χ^2^ test, *P* < .01

ERAS = enhanced recovery after surgery.

* Cases with atelectasis alone or combined with other complications.

† Cases with pleural effusion alone or combined with other complications.

‡ Cases with pneumonia alone or combined with other complications.

§ Cases with at least 1 type of complication (no double counting).

Notably, significant decreases were observed in 2 common postoperative complications following thoracic surgery such as the atelectasis and pleural effusion. The incidence of atelectasis was 15.6% in the ERAS group and 37.5% in the conventional group (*P* < .05), while the incidence of pleural effusion was 21.9% and 43.8%, respectively (*P* < .05). These reductions underscore the clinical relevance of key ERAS components such as structured respiratory training, early mobilization, and optimized analgesia.

Although the incidence of postoperative pneumonia did not differ significantly between groups (18.8% vs 28.1%, *P* > .05), the overall decline in major postoperative complications highlights the ability of the ERAS approach to mitigate surgical risk and promote safer recovery.

### 3.3. Pulmonary function recovery

A superior pulmonary function recovery was demonstrated in the ERAS group at both 1 week and 1 month after surgery (Table 5), underscoring the sustained protective effect of the ERAS intervention on respiratory performance. At 1 week postoperatively, the ERAS group exhibited a significantly higher mean FEV₁ compared with the conventional group (2.29 ± 0.68 L vs 1.38 ± 0.38 L, *P* < .001). Similarly, the mean FVC was markedly higher in the ERAS group (2.70 ± 0.87 L) than in the conventional group (1.68 ± 0.47 L, *P* < .001). This advantage in pulmonary function persisted at 1 month postoperatively. The mean FEV1 remained greater in the ERAS group (2.84 ± 0.74 L) than in the conventional group (2.01 ± 0.46 L, *P* < .01). The mean FVC was also significantly higher (3.26 ± 0.97 L vs 2.34 ± 0.60 L, *P* < .001).

**Table 5:** Pulmonary function (FEV1, FVC) at 1 week and 1 month postoperatively: absolute values and percentages of predicted values in both groups.

Time point	Group	FEV1 (L, % predicted)	Statistical method and *P* value (FEV1)	FVC (L), % predicted	Statistical method and *P* value (FVC)
1 wk	ERAS group (n = 32)	2.29 ± 0.68 L, 67.8 ± 14.2%	Independent samples *t*-test, *P* < .001	2.70 ± 0.87 L, 68.9 ± 13.8%	Independent samples *t*-test, *P* < .001
1 wk	Conventional group (n = 32)	1.38 ± 0.38 L, 42.7 ± 10.2%		1.68 ± 0.47 L, 45.1 ± 10.5%	
1 mo	ERAS group (n = 32)	2.84 ± 0.74 L, 83.9 ± 12.3%	Independent samples *t*-test, *P* < .01	3.26 ± 0.97 L, 83.3 ± 12.8%	Independent samples *t*-test, *P* < .001
1 mo	Conventional group (n = 32)	2.01 ± 0.46 L, 62.1 ± 10.9%		2.34 ± 0.60 L, 62.6 ± 11.0%	

ERAS = enhanced recovery after surgery, FEV1= forced expiratory volume in 1 second, FVC = forced vital capacity.

### 3.4. Imaging outcomes

Table 6 summarizes the imaging-based findings, which provide anatomical validation of the ERAS protocol’s efficacy and complement the earlier clinical and functional results. At 1 week postoperatively, patients in the ERAS group achieved a markedly higher lung re-expansion rate compared with those in the conventional group (89.2 ± 5.3% vs 72.6 ± 4.5%, *P* < .001). The ERAS cohort also exhibited a more favorable distribution of pleural effusion volumes. Specifically, 78.1% of ERAS patients presented with minimal effusions (<50 mL), compared with only 34.4% in the conventional group. In contrast, large effusions (>200 mL) occurred in only 3.1% of ERAS patients but in 18.7% of conventional patients – a statistically significant disparity (*P* < .001). These findings suggest that ERAS interventions effectively limit postoperative fluid accumulation and enhance pleural recovery. At 1 month postoperatively, the inflammatory resolution rate remained superior in the ERAS group (84.7 ± 5.9%) relative to the conventional group (68.0 ± 5.6%, *P* < .001).

**Table 6:** Imaging outcomes of postoperative lung re-expansion rate, pleural effusion volume and inflammatory resolution rate.

Outcomes	Groups	
	ERAS group (n = 32)	Conventional group (n = 32)
Lung re-expansion rate*	89.2 ± 5.3%	72.6 ± 4.5%
	Independent samples *t*-test, *P* < .001	
Pleural effusion volume†	<50 mL: 25 (78.1%); 50–200 mL: 6 (18.8%); >200 mL: 1 (3.1%)	<50 mL: 11 (34.4%); 50–200 mL: 15 (46.9%); >200 mL: 6 (18.7%)
	Chi-square test, *P* < .001	
Inflammatory resolution rate (%)‡	84.7 ± 5.9%	68.0 ± 5.6%
	Independent samples *t*-test, *P* < .001	

ERAS = enhanced recovery after surgery.

* Lung re-expansion rate measured at 1 week postoperatively.

† Pleural effusion volume measured at 1 week postoperatively.

‡ Inflammatory resolution rate measured at 1 month postoperatively.

### 3.5. Quality of life

At 1 month postoperatively, patients in the ERAS group reported significantly better HRQOL compared with those in the conventional group (Table 7). HRQOL was assessed using the EORTC QLQ-C30 questionnaire. The ERAS group achieved higher scores in both the total scale and the physical function subscale. The mean total score was 75.8 ± 5.4 in the ERAS group versus 58.9 ± 4.8 in the conventional group, while the mean physical function subscale score was 77.8 ± 5.4 versus 61.8 ± 4.8, respectively. Both differences were statistically significant (*P* < .001), indicating that the ERAS protocol not only accelerates physiological recovery but also enhances patients’ overall well-being and physical performance.

**Table 7:** Postoperative 1 month EORTC QLQ-C30 scores (total score and physical function subscale score) in the ERAS group and conventional group.

Score type	ERAS group (n = 32) Mean ± SD	Conventional group (n = 32) Mean ± SD
EORTC QLQ-C30 total score	75.8 ± 5.4	58.9 ± 4.8
Statistical method and *P* value	Independent samples *t*-test, *P* < .001	
EORTC QLQ-C30 physical function subscale score	77.8 ± 5.4	61.8 ± 4.8
Statistical method and *P* value	Independent samples *t*-test, *P* < .001	

EORTC QLQ-C30 = European Organization for Research and Treatment of Cancer Quality of Life Questionnaire-Core 30, ERAS = enhanced recovery after surgery, SD = standard deviation.

## 4. Discussion

This study demonstrates that a multimodal ERAS strategy, which integrates targeted perioperative analgesia, structured respiratory training, and early mobilization, significantly improved postoperative outcomes in patients undergoing VATS. These findings are consistent with prior evidence supporting thoracic ERAS protocols while addressing an important knowledge gap by incorporating objective imaging-based evaluations.

Consistent with van Haren et al,^[4]^ who reported reduced pulmonary and cardiac complications following ERAS implementation, our study found a 40.7% reduction in the total postoperative complication rate (31.2% vs 71.9%, *P* < .01), accompanied by significant declines in atelectasis (15.6% vs 37.5%, *P* < .05) and pleural effusion (21.9% vs 43.8%, *P* < .05). These improvements were accompanied by markedly shorter chest tube durations (2.84 ± 0.68 vs 5.44 ± 1.05 days, *P* < .01) and reduced postoperative hospital stays (4.50 ± 0.76 vs 7.38 ± 1.13 days, *P* < .01), paralleling the findings of Sedighim et al,^[23]^ who observed ERAS-associated reductions in length of stay following VATS lobectomy. Importantly, the ERAS group maintained superior pulmonary function at 1 month, with higher FEV1 (2.84 ± 0.74 vs 2.01 ± 0.46 L, *P* < .01) and FVC (3.26 ± 0.97 vs 2.34 ± 0.60 L, *P* < .001), confirming that ERAS supports durable respiratory rehabilitation beyond the immediate postoperative phase.

The enhanced efficacy of the ERAS protocol can be attributed to the synergistic effects of its core components, each addressing key barriers to recovery after VATS.

First, targeted perioperative analgesia – involving intraoperative intercostal nerve blocks with ropivacaine combined with optimized PCA – effectively mitigated persistent chest wall pain, a major obstacle to early mobilization and effective respiration in VATS patients, as highlighted by Bertolaccini et al.^[11]^ The significantly lower NRS pain scores observed in the ERAS group at postoperative day 1 (2.34 ± 0.48 vs 5.06 ± 0.88, *P* < .001) and day 3 (1.31 ± 0.47 vs 3.06 ± 0.88, *P* < .001) reduced opioid requirements and associated side effects – such as respiratory depression and ileus – thereby facilitating earlier ambulation. This finding aligns with Lee et al, who reported that nerve block–based analgesia reduced atelectasis rates among VATS patients by enhancing deep breathing and effective coughing.^[26]^

Second, structured respiratory training, initiated preoperatively and continued postoperatively, preserved respiratory muscle strength and promoted lung expansion. The ERAS group’s higher 1 week lung re-expansion rate (89.2 ± 5.3% vs 72.6 ± 4.5%, *P* < .001) reflects the benefit of diaphragmatic breathing and guided coughing, which maintain lung compliance and prevent focal atelectasis. This mechanism also explains the ERAS group’s favorable pleural effusion distribution (78.1% with minimal effusion < 50 mL vs 34.4% in the conventional group, *P* < .001), as improved lung expansion enhances pleural lymphatic drainage in line with the 2022 ESTS guidelines.^[24]^

Third, standardized early mobilization – with patients encouraged to ambulate at least 50 m within 24 hours postoperatively – worked synergistically with analgesia and respiratory training to reduce venous stasis, promote lung aeration, and minimize postoperative complications. This approach contributed to shorter hospital stays and faster recovery. In contrast, delayed mobilization, characteristic of conventional care, has been associated with prolonged chest tube retention and increased infection risk, as documented by Marques et al.^[6]^

A key innovation of this study lies in the systematic integration of imaging-based evaluation to validate the efficacy of the ERAS protocol, which addresses the historical reliance on subjective endpoints in thoracic ERAS research.^[6]^ Quantitative CT-derived lung re-expansion rates provided objective evidence of ERAS benefits: the ERAS group had a significantly higher 1 week rate and superior 1 week FEV1 than the conventional group, thereby confirming that anatomical recovery underpins functional improvement. This finding helps resolve the discrepancy reported by Ufuk et al, who noted that conventional clinical assessments failed to detect 47% of subclinical thoracic abnormalities. Moreover, chest radiography–based inflammatory resolution rates (84.7% vs 68.0% at 1 month, *P* < .001) further validated the sustained anti-inflammatory effects of ERAS – an important observation given that persistent pulmonary inflammation delays tissue repair and impairs long-term quality of life, as shown by Pfeuty et al.^[22]^ Imaging also proved valuable for clinical decision-making: CT-based assessment of pleural effusion volume reduced unnecessary reinterventions in the ERAS cohort, aligning with the 2024 ERAS consensus advocating for objective recovery markers.^[14]^

This study offers several strengths. Focusing exclusively on VATS, the most prevalent minimally invasive thoracic approach, enhances its clinical relevance. The integration of clinical, functional, and imaging outcomes provides a comprehensive evaluation of ERAS efficacy, while standardized surgical techniques and blinded assessments minimize potential bias. Nonetheless, several limitations should be acknowledged. The single-center retrospective design introduces possible selection bias, and the sample size of 32 patients per group may be underpowered to detect rare complications such as pneumonia, which showed no significant difference. Furthermore, the 1 month follow-up limits assessment of long-term outcomes, and the absence of opioid consumption data precludes a complete evaluation of ERAS-related analgesic benefits.

Clinically, these findings support routine incorporation of multimodal ERAS strategies into standard VATS care. Core interventions should be prioritized, including intraoperative intercostal nerve block, preoperative respiratory training, and early postoperative mobilization, particularly for patients with compromised pulmonary reserve. Routine chest CT at 1 week postoperatively is recommended to guide chest tube removal and detect subclinical abnormalities, thereby reducing unnecessary hospitalization. Future studies should employ multicenter prospective randomized controlled trials to confirm efficacy across diverse populations, extend follow-up to 6 to 12 months to capture long-term outcomes, and explore personalized ERAS optimization through preoperative imaging-based risk stratification.

## 5. Conclusion

This study evaluated a multimodal ERAS strategy for elective VATS patients, integrating clinical, functional, and imaging outcomes. The results show that targeted analgesia, structured respiratory training, and early mobilization significantly improve recovery, reduce complications, preserve pulmonary function, and enhance quality of life at 1 month postoperatively. Imaging provides objective confirmation of lung recovery and complements traditional clinical assessments. This multimodal ERAS strategy should be adopted as a standard perioperative care approach for VATS patients to optimize clinical outcomes and the use of healthcare resources.

## Author contributions

**Conceptualization:** Xinye Li, Zhenjun Hu, Aotian Guo, Zhengfu He.

**Data curation:** Xinye Li, Zhenjun Hu, Aotian Guo, Zhengfu He.

**Formal analysis:** Xinye Li, Zhenjun Hu, Aotian Guo, Zhengfu He.

**Funding acquisition:** Xinye Li, Zhengfu He.

**Investigation:** Xinye Li, Zhengfu He.

**Writing – original draft:** Xinye Li, Aotian Guo, Zhengfu He.

**Writing – review & editing:** Xinye Li, Aotian Guo, Zhengfu He.
